# Mitochondrial Behavior in Axon Degeneration and Regeneration

**DOI:** 10.3389/fnagi.2021.650038

**Published:** 2021-03-08

**Authors:** Biyao Wang, Minghao Huang, Dehao Shang, Xu Yan, Baohong Zhao, Xinwen Zhang

**Affiliations:** ^1^The VIP Department, School and Hospital of Stomatology, China Medical University, Liaoning Provincial Key Laboratory of Oral Diseases, Shenyang, China; ^2^Center of Implant Dentistry, School and Hospital of Stomatology, China Medical University, Liaoning Provincial Key Laboratory of Oral Diseases, Shenyang, China

**Keywords:** mitochondria, axon regeneration, aging, traumatic brain injury, spinal cord injury, Alzheimer's disease, Parkinson's disease, Amyotrophic lateral sclerosis

## Abstract

Mitochondria are organelles responsible for bioenergetic metabolism, calcium homeostasis, and signal transmission essential for neurons due to their high energy consumption. Accumulating evidence has demonstrated that mitochondria play a key role in axon degeneration and regeneration under physiological and pathological conditions. Mitochondrial dysfunction occurs at an early stage of axon degeneration and involves oxidative stress, energy deficiency, imbalance of mitochondrial dynamics, defects in mitochondrial transport, and mitophagy dysregulation. The restoration of these defective mitochondria by enhancing mitochondrial transport, clearance of reactive oxidative species (ROS), and improving bioenergetic can greatly contribute to axon regeneration. In this paper, we focus on the biological behavior of axonal mitochondria in aging, injury (e.g., traumatic brain and spinal cord injury), and neurodegenerative diseases (Alzheimer's disease, AD; Parkinson's disease, PD; Amyotrophic lateral sclerosis, ALS) and consider the role of mitochondria in axon regeneration. We also compare the behavior of mitochondria in different diseases and outline novel therapeutic strategies for addressing abnormal mitochondrial biological behavior to promote axonal regeneration in neurological diseases and injuries.

## Introduction

Mitochondria are dynamic organelles playing a pivotal role in energy generation, signaling, and calcium homeostasis (Han et al., 2016). Axonal mitochondria exhibit a linearly interspersed distribution. Approximately 87% of mitochondria are stationary, and the number of motile mitochondria decreases from the proximal to distal axon (Cheng and Sheng, 2020). Mitochondria are transported to specific sites in axons where high energy is in demand, such as in growth cones and axonal branches (Morris and Hollenbeck, 1993; Tao et al., 2014). These distribution events are stabilized when presynaptic structures and mature neurons are formed. Moreover, mitochondria will rapidly redistribute in response to physiological or pathological stress in order to maintain energy homeostasis. The vigorous response of axonal mitochondria redistribution is predominantly conducted by the cooperation of microtubule-based transport and anchoring substrates, including anchors, adaptors, and motors (Sheng, 2014, 2017; Misgeld and Schwarz, 2017; Guedes-Dias and Holzbaur, 2019). Impairment of these substrates and process will result in the inhibition of axonal mitochondrial transport and ultimately induce local energy depletion and axon degeneration (Sheng and Cai, 2012).

Axon degeneration has been observed during normal neuronal aging, injury (e.g., traumatic brain injury, TBI; spinal cord injury, SCI; Maxwell, 2015; Wang et al., 2019b) and neurodegenerative diseases (e.g., Alzheimer's disease, AD; Parkinson's disease, PD; Amyotrophic lateral sclerosis, ALS; Guo et al., 2020). Axon degeneration usually occurs before the neuronal soma's death at the early stage of the diseases. In most conditions above, there are two different processes of axon degeneration. The distal axons won't connect to soma undergoing Wallerian degeneration while the proximal axons will die back toward soma (Adalbert and Coleman, 2013; Gerdts et al., 2016). Axon degeneration has an intimate association with mitochondrial dysfunction, including energy deficits, oxidative stress, disequilibrium of mitochondrial fission and fusion, impaired axonal mitochondrial transport, and aberrant mitophagy (Court and Coleman, 2012; Sheng and Cai, 2012; Geden and Deshmukh, 2016). Therefore, analysis of the mitochondrial behavior in axons may be considered a novel target for therapeutic strategies.

The intrinsic capacity and process of axon regeneration are different between the peripheral and central nervous systems (PNS and CNS, respectively), and the capacity of the former is superior to that of the latter (Fawcett and Verhaagen, 2018; Mahar and Cavalli, 2018). Axon regeneration in PNS initiates a vigorous regenerative response via the permissive Schwann cell environment and form a new growth cone. This process is supported by a regenerative program through expression of the Regeneration-associated gene (Attwell et al., 2018; Mahar and Cavalli, 2018). Nevertheless, axotomy at the central branch has a minimal regenerative response and the Regeneration-associated gene response also seems to make no difference on axon regeneration (Chandran et al., 2016; Cartoni et al., 2017). While in the CNS, the ability of axon regeneration is dependent on the maturity of the neurons. Moreover, the environment of the injured neuron is full of molecules and structures which may suppress axon regeneration in CNS (Fawcett and Verhaagen, 2018). CNS axons cut from embryos exhibits a potential to grow for long distance after transplanted into an adult CNS via expressing a series of molecules inducing axon growth (Reier et al., 1986; Lu et al., 2012). Nevertheless, axons from the adult CNS respond to axotomy with a minimal regenerative response and a poor change of gene expression despite being in a permissive environment. These diverse response to injury in PNS and CNS (mature and immature) form the concept of intrinsic regenerative capacity of axon regeneration (Fawcett and Verhaagen, 2018). Because this process consumes a large amount of energy and molecules or substrates during restoration, alteration of mitochondria behavior may represent one of the prominent intrinsic regenerative capacities in axon regeneration; however, the mechanisms underlying this process have just begun to be elucidated.

In this review, we summarize the normal mechanism of mitochondrial quality control in axons. We also describe the behavior of mitochondria in axon degeneration under different conditions (i.e., aging, nerve trauma, and neurodegenerative diseases) and their role in axon regeneration and the treatment methods derived from these effects.

## Normal Mitochondrial Behavior in Axons

Mitochondrial transport is indispensable for mitochondrial quality control. Anterograde transport contributes to the energy supply and neuronal survival, and retrograde transport helps with damaged mitochondrial clearance (Chen et al., 2016). Only a small proportion of axonal mitochondria (30–40%) is motile *in vivo* and *in vitro*; most mitochondria are stalled and do not move for long periods (Zheng et al., 2019). Mitochondrial transport depends on interactions between microtubules, microtubule motor proteins, adaptor complexes that bind the motor proteins to mitochondria, and their anchors.

Mitochondria are transported along the microtubule filament by the motor proteins kinesin and dynein to the plus and minus ends, respectively (Lin and Sheng, 2015). Kinesin-1 (KIF5) is the major motor protein involved in anterograde transport, the other member of kinesin family such as KIF1Ba and KLP are also participants (Zheng et al., 2019). Mitochondria interact with motor proteins via the adaptor complex formed by Miro, a GTPase located in the outer mitochondrial membrane (OMM), and Milton/TRAK (TRAK 1 and 2 in mammals). TRAK1 binds both the heavy chain of KIF5 and dynein; however, TRAK2 preferentially binds dynein (Smith and Gallo, 2018). Compared to the preference for dynein in dendrites, axonal mitochondria bind either kinesin or dynein via the Miro-TRAK1 complex.

The axon-specific mitochondrial outer membrane protein syntaphilin (SNPH) is a representative cytoskeletal tether (i.e., anchor), which opposes motility by increasing the force between the mitochondria and microtubules and inhibiting ATPase activity (Chen and Sheng, 2013). Microtubule-associated protein, such as myosin V or myosin VI (Pathak et al., 2010), and actin filament-based mechanisms may also regulate stalling and transport in axons (Henderson et al., 2017). Cytosolic calcium is another key regulator of mitochondrial transport. There are two main mechanisms for calcium resulting in the stalling of mitochondria (Zheng et al., 2019). For one thing, SNPH can bind kinesin I and sequester motor protein in the context of calcium signaling. For another, Miro has two EF calcium binding domains and Miro will directly bind KIF5 when binding of calcium (Liu and Hajnóczky, 2009), which will prevent motor activity. Therefore, increased intracellular calcium levels are sufficient to inhibit this process (Woolums et al., 2020).

A dynamic process in mitochondria (i.e., mitochondrial fission and fusion) continuously occurs to maintain a steady mitochondrial morphology and population (Yan et al., 2020b). Mitochondrial fission helps a damaged mitochondrion segregate from the healthy one while mitochondrial fusion facilitates the interaction and fusion of two mitochondria, which repairs each other, demonstrating that the equilibrium of fission and fusion acts a pivotal part in axonal mitochondria (Yu et al., 2016; Pickles et al., 2018). Mitochondrial fission is mediated by the GTPase dynamin-related protein 1 (Drp1), which is recruited to the OMM and forms a contractile ring around the mitochondrion, dividing one mitochondrion into two separate mitochondria (Otera et al., 2013). GTPase dynamin-related protein 1 (Drp1)-independent mitochondrial fission can also occur (Rival et al., 2011; Stavru et al., 2013; Roy et al., 2016). Mitochondrial fusion involves two separate processes, the fusion of the OMM, which is regulated by mitofusins (Mfns), and the fusion of the inner mitochondrial membrane that is mediated by optic atrophy 1. The fusion of the OMM is regulated by mitofusins 1 and 2 (Mfn1 and Mfn2). Both mitochondrial fission and fusion take place along the axon (Yan et al., 2020b).

Mitophagy plays a key role in relieving oxidative stress and preventing axonal and cytosolic defects and subsequent cell death (Wang et al., 2018). To maintain mitochondrial quality control in axons and cells, mitophagy degrade and recycle damaged mitochondria undergoing physiological or pathological processes, such as mitochondria depolarization and oxidative stress (Martinez-Vicente, 2017). The PINK1/Parkin pathway is a canonical mechanism of mitophagy. In addition to the PINK1/PARKIN pathway, other OMM proteins directly interact with LC3 and promote mitophagy in mammals, including FUNDC1, NIX/BNIP3L, and Bcl2-L-13 (Di Rita et al., 2018).

## Aging

### Mitochondrial Behavior in Axon Degeneration in Aging

Aging is a normal physiological phenomenon, and glaucoma and many neurodegenerative diseases (e.g., Alzheimer's disease, Parkinson's disease) are related to aging. Mitochondrial behavior plays a key role in axon degeneration. First, aging has been shown to be associated with mitochondrial transport. Glaucoma is a chronic and progressive neurodegenerative disease. Degeneration of ganglion cells and their axons has been shown to be related to aging (Liu et al., 2018). In ganglion cell neurons, with age the cross-section axon area enlarges characterized by axoplasm disorganization and accumulation of hyperphosphorylated neurofilaments which is indicative of axonopathy (Cooper et al., 2016). Further, there are more mitochondria-free regions and decreased lengths of mitochondrial transport in degenerative axons (Mao et al., 2016). The changes in these mitochondria with age are related to changes in the expression of Mfn2. Among the healthy mice in the control group, the level of Mfn2 in retina was slightly elevated in aged mice (Nivison et al., 2017). Considering the function of Mfn2 in maintaining mitochondrial morphology and participating in mitochondrial transport (Misko et al., 2010), this increase indicates the demand of mitochondrial fusion and transport. The increase was more pronounced in retina of DBA mice (a murine glaucoma model), which explained the mitochondria in glaucoma neurons were elongated or fused. However, Mfn2 is either damaged or cannot be transported to optic nerve, resulting in a decrease in the level of this protein in optic nerve, indicating that Mfn2 protein is not transported down axons to function in mitochondrial transport and repair, which leads to disease progression. Moreover, given that phosphorylated Mfn2 is a receptor for Parkin on depolarized mitochondria, the amount of phosphorylated Mfn2 in young mice are also higher than the aged control indicating that the mitochondria transport is altered (Nivison et al., 2017).

Pathological morphology of mitochondria has been reported in degenerative axons with age. The average diameter of axonal mitochondria increases, with few or no cristae observed in the aging axonal mitochondria (Cao et al., 2017). This phenomenon is attributed to reduced optic atrophy 1 and Mfn2 expression (Rebelo et al., 2018) and even the absence of expression at the aging axon terminal. This morphological change is also relevant to neuronal apoptosis, P53-dependent neuronal cell death is associated with increased mitochondrial length mediated by reduced Drp1 and Parkin expression during aging (Wang D. B. et al., 2013).

Different phenomena are observed for mitophagy in axons *in vivo* and *in vitro*. *In vitro*, Sung et al. (2016) demonstrated that autophagosomes co-localized with mitochondria in axons when neurons were treated with drugs to induce mitochondrial damage. However, evidence from *in vivo* imaging suggests that either PINK1/Parkin or Parkin-dependent or mitophagy is indispensable for mitochondria turnover and axon integrity (Cao et al., 2017). Further research is needed to determine whether the mitochondria in axons need to undergo mitophagy or are transported back to the soma.

Finally, all of the described changes in mitochondrial behavior may affect bioenergetics by influencing oxidative phosphorylation and producing excessive reactive oxidative species (ROS). The molecular mechanisms regulating this process in aging include increases in the levels of proteins related to mitochondria and oxidative phosphorylation, more specifically, Complex I (Kline et al., 2019).

### Mitochondrial Behavior in Axon Regeneration in Aging

The relationship between aging and axon regeneration is complex and affected by multiple factors. Both endogenous (Geoffroy et al., 2017) and exogenous factors (Sutherland and Geoffroy, 2020) of neurons play an important role in the ability of axons to regenerate after damage and this process is declining with aging (Geoffroy et al., 2016). Although young neurons have a strong ability for axon growth, mature neurons can usually not regenerate after injury because mature CNS axons lose their ability to regenerate due to most mitochondria being stationary, which is associated with high SNPH expression (Lewis et al., 2016). Therefore, without enough local mitochondria, sufficient ATP cannot be provided, and the process of axon regeneration, including re-sealing cell membranes, rearranging cytoskeletal structures, and reforming active growth cones, will be hindered (Bradke et al., 2012). However, mitochondrial transport can be enhanced by stimulating the cAMP/Protein Kinase A (PKA) pathway to upregulate kinesin-1 expression to counteract its decrease during aging (Vagnoni and Bullock, 2018). Interestingly, the effects of cAMP or PKA on increasing mitochondrial transport were more pronounced in the aging group than in the younger group.

The internal components of the mitochondria also affect axonal regeneration by modifying the biological behavior of mitochondria and oxidizing the respiratory chain. In addition to acting as a transcription factor for axon regeneration in the nucleus, STAT3 can also reach the inner mitochondrial membrane following the activation of mitogen-activated protein kinase (MAPK), which improves axon regeneration by enhancing cell bioenergetics in the spinal cord (Luo et al., 2016) in mature mice with a reduced regenerative capacity. The mitochondrial signature phospholipid cardiolipin present in the inner leaflet of the inner mitochondrial membrane is essential for mitochondrial dynamics, mitochondrial biogenesis, and energy metabolism in the mitochondria (Mårtensson et al., 2017).

## Injury

### Traumatic Brain Injury

Traumatic brain injury (TBI) leads to the dynamic deformation of the parenchyma, resulting in shear and stretch injuries to axons, commonly known as diffuse axonal injury. Diffuse axonal injury is demonstrated to result from the mechanical deformation of the axonal cell membranes via calpain-mediated proteolysis of sidearms or phosphorylation, triggering neurofilament compaction, calcium changes, microtubules destabilization, and metabolic dysfunction (Barkhoudarian et al., 2016). Subsequently, axoplasmic transport mechanisms are defective, which leads to the accumulation of transport products that cause axonal swelling, secondary disconnection, and Wallerian degeneration (Frati et al., 2017).

#### Mitochondrial Behavior in Axon Degeneration Following TBI

Transmembrane sodium channels are damaged in TBI, leading to an abnormal influx of calcium into the axonal plasma (Maxwell, 2015). Calcium is also released from mitochondria and the axoplasmic reticulum (Springer et al., 2018), leading to axonal mitochondria dysfunction. Under Transmission electron microscopy, the axoplasm contains damaged or lucent mitochondria and a disorganized cytoskeleton within injured nerve fibers (Maxwell, 2015). The axonal swellings of TBI are characterized by the aggregation of membranous organelles, especially mitochondria, resulted from the injury and loss of axonal microtubules, which interact with dynein and kinesin (Maxwell, 2015). It is reported that the loss of axonal microtubules in TBI results in the lack of neuronal mitochondria delivery, failing to replace those damaged by the destroyed calcium homeostasis and to provide vesicular profiles (Maxwell et al., 1997; Tuck and Cavalli, 2010). Within an injured mouse optic nerve fiber, which is far away from the secondary axotomy site, accumulating evidence demonstrated the dieback or withdrawal of the proximal and distal terminal swellings (Staal et al., 2010; Wang et al., 2011). In the latter study, the proximal swellings contained intact mitochondria while the distal swellings exhibited defected mitochondria short of cristae, which appeared consistent with an ongoing degenerative process (Wang et al., 2011). In addition, the distal swelling exhibits three types of mitochondria, including mitochondria aggregating pyroantimonate crystals, those unremarkable or intact, and those containing electron lucent spaces in the mitochondrial matrix (Wang et al., 2011).

Damaged mitochondria fail to generate adequate ATP and cannot support the essential physiological and biochemical processes and disturb the transmembrane transporter function (Maxwell, 2015). Furthermore, oxidative stress in TBI results from the uncontrolled influx of Ca^2+^, leading to calcium accumulation in the mitochondria. The subsequent activation of caspases and calpains correlates with the initiation of apoptosis (Frati et al., 2017). Calpain-mediated spectrin proteolysis reaction product, which is reported to be correlated with damaged mitochondria, spread widely in the axoplasm at foci of neurofilament compaction (Büki et al., 1999), suggesting the defection of neurofilaments (Dewar et al., 2003). Moreover, the release of cytochrome c in the damaged axons triggers retrograde signaling by “apoptosis-inducing factor,” resulting in the programmed cell death and apoptosis of injured neurons (Büki et al., 2000).

#### Mitochondrial Behavior in Axon Regeneration Following TBI

Mitochondrial transport and bioenergetics are enhanced to facilitate axon regeneration following TBI (Misgeld et al., 2007). Within axon regeneration in TBI, it is beneficial to elevate the density of mitochondria while decreasing the density is disadvantageous. Mitochondria are transported into the injured zone in order to elevate the average mitochondria density. Regenerating axons have a higher density of mitochondria than non-regenerating axons in *Caenorhabditis elegans* (Han et al., 2016). Accumulating evidence in the SNPH knockout mice demonstrates that mitochondrial transport is strengthened to clear impaired mitochondria, replenish healthy mitochondria in injured axons, and ultimately reverse the energy deficits in axon regeneration (Zhou et al., 2016; Cheng and Sheng, 2020). In contrast, the Armadillo Repeat Containing X-Linked 1 protein, responsible for connecting the Miro adaptor protein with the mitochondrial membrane, is overexpressed, leading to the acceleration of optic nerve regeneration following TBI (Cartoni et al., 2016). Moreover, the dual leucine zipper kinase 1 microtubule-associated protein kinase pathway in *C. elegans* can promote adequate mitochondrial transport into the regeneration zone independent of Miro to increase the mitochondria density in injured axons (Han et al., 2016). Overall, axon regeneration following TBI can be achieved by increasing the density of mitochondria by enhancing the transport of healthy mitochondria, which provide sufficient energy for axonal regrowth.

### Spinal Cord Injury

The damage caused by SCI is mainly divided into two stages. The initial trauma is mainly caused by a contusion or direct compression. The secondary injury is caused by disruption of blood vessels, microcirculation failure, ion homeostasis disorder, excessive production of free radicals, and inflammation (Wang S. et al., 2019).

#### Mitochondrial Behavior in Axon Degeneration Following SCI

After acute SCI and the accompanying depolarization of neurons, mitochondrial permeability transition pores are opened by calcium influx, which impairs ATP synthesis, destroys the mitochondrial outer membrane, and releases ROS and pro-apoptotic proteins into the cytoplasm (Pivovarova and Andrews, 2010). In an SCI rat model, swollen and disrupted mitochondria with disorganized cristae can be observed in both pre-apoptotic neurons and astrocytes (Xu et al., 2017).

Immunofluorescence staining of post-mortem tissue from *Thy1YFP*+ transgenic mice showed that a significant increase in TOMM20 signal reflected the density of mitochondria in the axonal spheroids and endbulbs (Rajaee et al., 2020). The accumulation of mitochondria and tubulin polyglutamylation indicates the disruption of axonal transport, an important characteristic of axonal degeneration. Moreover, the failure of mitochondria to migrate to the remote ends of axons indicates abnormal mitochondrial axonal localization that contributes to axon retraction.

In the early stage of SCI, mitochondria fuse to restore the respiratory chain and inhibit mitochondrial apoptosis. In contrast, mitochondrial fission resulting from mitochondrial dysfunction further inhibits microtubule stabilization and axonal regeneration and is associated with apoptosis (Csordás et al., 2018). The dysfunction of mitochondrial fission and fusion caused by SCI is associated with the upregulation of Drp1, which reduces mitochondrial membrane potential, releases cytochrome C and caspase3, and induces neuronal apoptosis (Jia et al., 2016).

#### Mitochondrial Behavior in Axon Regeneration Following SCI

Within a few days to a few weeks after a person suffers axon damage, the damaged spinal cord begins to recover. To solve the functional defect caused by the separation of the white matter tracts between the rostral and caudal spinal cords, axonal regeneration is required to reconnect the caudal/rostral neurons and form new neural circuits to restore signal conduction (O'Shea et al., 2017). However, observations in mice showed that the reduced growth capacity of mature neurons and scar tissue caused by reactive astrocytes form a physical barrier, and the upregulation of axon growth-inhibitory factors inhibit axon regeneration (Yiu and He, 2006). However, intravital imaging of murine spinal cord showed that the number of axons increases significantly from 7 to 14 days after injury compared to 3 days post-injury, indicating that a certain number of injured axons undergo endogenous repair (Rajaee et al., 2020), demonstrating that while the regeneration of axons is limited after SCI injury, it can still be improved.

Given the important role of axonal mitochondria in growth cone migration, mitochondrial transport is required for axonal regeneration after SCI. Spinal cord injury (SCI) itself does not directly affect mitochondrial transport in axons; however, enhancing the transport of mitochondria after SCI is helpful for this process. *In vitro* data showed that fibroblast growth factor-13 co-localizes with mitochondria, and its increased activity and density in axons may, in part, explain why fibroblast growth factor-13 promotes axon regeneration in SCI models (Li et al., 2018).

Changing mitochondrial dynamics after SCI, specifically by promoting mitochondrial fusion and inhibiting its fission, positively affects axon regeneration. In an SCI model, Loureirin B significantly increased both bcl-2 and Mfn1 and doubled the size of the mitochondria, which promoted fusion and facilitated axon regeneration (Wang et al., 2019a). Considering the adverse effects of Drp1 upregulation after SCI injury, mitochondrial division inhibitor-1, an inhibitor of Drp1, promotes animal recovery and reduces apoptosis (Jia et al., 2016).

### Peripheral Nerve Injury

Once the peripheral nerve is injured, the axon is divided into two segments; the proximal axon segment and the distal segment. The distal axon undergoes Wallerian degeneration, while the proximal axon possibly regenerates via a growth cone, triggering axon extension (Neukomm et al., 2017).

#### Mitochondrial Behavior During Axon Degeneration Following Peripheral Nerve Injury

In the injured peripheral neuronal axon of *C. elegans* and ric-7 (resistant to inhibitors of cholinesterase) mutants, the density of healthy mitochondria is decreased while the density of defected mitochondria is elevated, leading to energetic failure and oxidative stress (Rawson et al., 2014). Furthermore, mitochondrial transport is similarly inhibited in the Thy1-mitoCFP transgenic mouse (Thy1-MitoCFP mice expressing CFP fused to the human cytochrome c oxidase mitochondrial targeting sequence were from Jackson Laboratories; Misgeld et al., 2007).

In response to injury, multiple proteins are inhibited or activated during distal axon degeneration. Together with increased calcium influx, decreased nicotinamide mononucleotide adenyltransferase (NMNAT) and NAD1 activates SARM1 (Sterile Alpha and TIR Motif Containing 1), which are key initiating factors in Wallerian degeneration (Osterloh et al., 2012). Sterile Alpha and TIR Motif Containing 1 (SARM1) activation results in mitochondrial dysfunction, calpain-dependent defects in the cytoskeleton, and subsequent axon degeneration (Adalbert et al., 2012; Ma et al., 2013; Yang et al., 2015). Wallerian degeneration slow fusion protein (WldS), comprising full NMNAT1, has been shown to alleviate degeneration in Zebrafish and Drosophila (Coleman et al., 1998; Conforti et al., 2000). NMNAT1 has a pivotal role in maintaining axon integrity (O'Donnell et al., 2013; Wang Y. et al., 2015), the activity of which is greatly inhibited by defects in healthy mitochondria, decreases in mitochondrial transport, and ROS accumulation during axon degeneration (Yahata et al., 2009; Avery et al., 2012; Fang et al., 2012; O'Donnell et al., 2013). The dual leucine zipper kinase (DLK) is a mitogen activated protein kinase (MAP3K), and contributes to axon degeneration by reducing energy production. Dual leucine zipper kinase (DLK) is an axonal integrity sensor and interacts with NMNAT and activated SARM1 (Cavalli et al., 2005; Xiong et al., 2012; Yang et al., 2015; Gerdts et al., 2016); subsequently, SARM1 interacts with Axundead to consume NAD1 (Essuman et al., 2017; Neukomm et al., 2017). Decreased NAD1 production reduces energy generation and ATP levels, causing defects in Na^+^/Ca^2+^ exchangers and Ca^2+^ channels, and the loss of mitochondrial membrane potential (Gerdts et al., 2015; Loreto et al., 2015). Thus collectively, these proteins damage axon integrity.

#### Mitochondrial Behavior During Axon Regeneration Following Peripheral Nerve Injury

In contrast to the central nerve, after peripheral nerves are injured, axons have a strong regenerative ability to restore function. At the proximal segment of peripheral nerve injury, Golgi-derived vesicles transport anterogradely to aggregate near the axon end to repair the ruptured membrane, while increasing intracellular Ca^2+^ levels activate kinases and phosphatases assemble microtubules to support the formation of new growth cones, and drive axon elongation (Girouard et al., 2018). This process is relevant to Schwann cells (Jessen et al., 2015; Nocera and Jacob, 2020) and intrinsic signaling events induced by injury, such as growth-associated protein-43 (GAP-43) (Chung et al., 2020).

Mitochondria are essential for axon regeneration. Increased mitochondrial density in *C. elegans* effectively promotes axon regeneration (Han et al., 2016). Reduced oxidative stress and enhanced axon regeneration are observed in a sciatic nerve crush injury model when mitochondria are injected into injured nerves (Kuo et al., 2017). Thus, increasing mitochondrial density at the site of injured axons can be achieved, mainly by increasing mitochondrial transport in axons, and increasing mitochondrial fission. Miro overexpression enhances mitochondrial transport in proximal segments, whereas axon regeneration is significantly inhibited in individuals where Miro is suppressed (Han et al., 2016). In addition, by imaging axonal mitochondrial transport *in vivo*, and when the intercostal nerve is damaged, mitochondrial anterograde transport is increased (Misgeld et al., 2007), which may be associated with tyrosinated tubulin, and upregulated molecular motors (Yang et al., 2021). An *in vivo* sciatic nerve compression study indicates that enhanced mitochondrial transport, via SNPH knockout in mice, accelerates axon regeneration in the peripheral nervous system (Zhou et al., 2016). The early fission of mitochondria after axon damage is also associated with axon regeneration. For example, in mice, sciatic nerve transection generates significantly increased mitochondrial division in damaged axons (Kiryu-Seo et al., 2016). This is further confirmed by the inhibition of axon regeneration in individuals with mutations in the mitochondrial fusion gene, OPA1 (Knowlton et al., 2017).

Mitochondrial regeneration after peripheral nerve injury highlights the importance of axon transport, to not only provide structural components of cells such as lipid vesicles and microtubules, but to meet the high energy requirements of regenerating axons (Prior et al., 2017; Mahar and Cavalli, 2018). In an *in vitro* sciatic nerve injury study, Hwang et al. found that after sciatic nerve injury, axon CDK5 levels increased, they were translocated into the mitochondria and phosphorylated mitochondrial STAT3, thereby regulating mitochondrial activity (Hwang and Namgung, 2021). STAT3 phosphorylation affects microtubule assembly and energy production in axons (Selvaraj et al., 2012). When translocated to the mitochondria, STAT3 interacts with electron transport chain proteins, regulating ROS production and the release of cytochrome C (Szczepanek et al., 2011), and contributing to ATP energy supply to promote axon regeneration.

### Treatment

Mitochondria in the injured axon have pivotal roles in axon degeneration and regeneration. Therefore, mitochondria may be specific therapeutic targets for inhibiting axon degeneration and enhancing axon regeneration. Oxidative stress caused by dysfunctional mitochondria results in a variety of adverse effects. Thus, a potential therapeutic strategy would be to decrease this stress. In a TBI animal model, cyclosporin, an anti-oxidative drug, can decrease cytochrome c release from mitochondria and inhibit Ca^2+^ influx into mitochondria, ultimately alleviating mitochondrial dysfunction and axon damage (Kelsen et al., 2019). Additionally, ROS-mediated axonal degeneration in TBI caused by the aberrant axonal calcium homeostasis is reported to be alleviated via the application of calcium channel blocker (McAllister, 2011; Namjoshi et al., 2014). Moreover, Metformin can stabilize the microstructure in injured axons of SCI by activating the PI3K/Akt signaling pathway. Furthermore, activation of Akt/Nrf2 signaling could be a potential approach for axon regeneration treatment by inhibiting excessive oxidative stress and restoring mitochondrial function (Wang et al., 2020).

Enhancing mitochondrial transport and recruitment aids axon regeneration in injured axons and facilitates the removal of damaged mitochondria and replenishment of healthy ones to provide adequate ATP (Kaasik, 2016; Sheng, 2017; Smith and Gallo, 2018). Inhibiting the microtubule-severing protein Fidgetin (Matamoros et al., 2019) or non-muscle myosin II (Hur et al., 2011) promotes axon regeneration after SCI by facilitating the reorganization of microtubule and actin cytoskeletal proteins. Moreover, Loureirin B promotes mitochondrial fusion and suppresses ER stress by activating the Akt/GSK-3β pathway, facilitating axon regeneration (Wang et al., 2019a). In summary, combined approaches targeting mitochondria may provide a novel therapeutic strategy for alleviating axon degeneration and enhancing axon regeneration in TBI and SCI.

## Neurodegenerative Disease

### Alzheimer's Disease

Alzheimer's disease is an age-related neurodegenerative disease characterized by progressive cognitive impairment, mobility disorder, and memory loss. Hallmarks of AD include aberrant amyloid-β (Aβ) metabolism and neurofibrillary tangles of hyperphosphorylated Tau protein, which are particularly significant in axon degeneration (Wang et al., 2017). β-site amyloid precursor protein (APP) cleaving enzyme 1 (BACE1) is the major neuronal Aβ-secretase for Aβ generation. Axon degeneration in AD involves a complicated mechanism, including glucose metabolism defects, mitochondrial dysfunction, aberrant calcium homeostasis, oxidative stress, and imbalances in energy homeostasis (Cieri et al., 2018; Mata, 2018; Swerdlow, 2018; Albensi, 2019).

Accumulating evidence has demonstrated that aggregation of pathological Tau and Aβ impairs mitochondria transport (anterograde and retrograde) in axons (Wang Z. X. et al., 2015; Sadleir et al., 2016; Cai and Tammineni, 2017; Zhang et al., 2018). One of the first researches in this area was carried out in primary neurons from *Tg2576* mice with mutant human APP protein (Calkins et al., 2011). Compared to wild-type neurons, primary neurons from *Tg2576* mice exhibited inhibited anterograde mitochondrial transport as well as promoted mitochondrial fission and inhibited mitochondrial fusion (Calkins et al., 2011). Mitochondria transport in axons requires a variety of substrates and molecular support, whose dysfunction can lead to negative impact on mitochondria transport. The defected transport results in the reduction of mitochondria density at the distal axons and the depletion of axonal NMAT protein 2 (NMAT2), which is continually supplemented in axons via fast axonal transport (Ljungberg et al., 2012; Ali et al., 2016). Following axotomy, the transport of NMNAT2 toward axons is inhibited, meanwhile, it has a rapid decline in axons before Wallerian degeneration occurs (Gilley and Coleman, 2010; Gerdts et al., 2016). Since the damaged mitochondria inhibited anterograde and promoted retrograde transport, the quantity of SNPH cargo vesicles is significant elevated in AD axons, similar to ALS. The SNPH-mediated response in axonal mitochondrial transport of AD-related cortical neurons from mutant human APP-expressing transgenic mice is summarized in the ALS section (Lin et al., 2017a,b; Cheng and Sheng, 2020). Ultimately, the impairment of mitochondrial axonal transport in AD inhibits OXPHOS complex activity, causing significant energy deficiency (Spires-Jones and Hyman, 2014).

These two AD hallmarks contribute to mitochondrial transport pathology in axons. Defects in axon transport can be initiated by soluble low molecular weight Aβ species in the plasma membrane in mice (Zhang et al., 2018). Moreover, inhibited axon transport does not promote mitochondrial dysfunction or ATP depletion at once (Zhang et al., 2018), suggesting that reconstruction of mitochondrial axon transport may be advantageous at early AD stages. The restoration of KIF5A, an isoform of kinesin-1, abrogates the impairment of mitochondria axonal transport by Aβ, particularly anterograde transport in 5 × FAD mice (Wang et al., 2019b). Additionally, it is reported that axon degeneration has an intimate correlation with mitochondrial dysfunction and mPTP (Barrientos et al., 2011). The deficiency of cyclophilin D (CypD), a mPTP regulator, inhibited Aβ-mediated permeability transition and mitochondrial swelling, and alleviated oxidative stress both in mice model and brain samples of AD. Notably, knockout of *CypD* gene appears to suppress the mPTP, which may improve cognitive and synaptic function in mouse model (Du et al., 2008, 2011). Moreover, the defection of mitochondrial axon transport triggered by Aβ is dependent on the activation of mPTP, which is mediated by *CypD*. Knockout of *CypD* inhibit the induction of the p38/MAPK signaling pathway mediated by Aβ, restore the dysfunction of axonal mitochondria and synapses (Guo et al., 2013). Furthermore, the defective dynein-snapin coupling mediated by Aβ seriously inhibits retrograde transport, resulting in the aggregation of amphisomes at axonal terminals, which can greatly contribute to mitophagy (Tammineni et al., 2017). Therefore, it seems that there is an intimate relationship between the impaired axonal transport and mitophagy, together consist of the complicated mechanism of mitochondrial dysfunction in axons of AD.

Overexpression or hyperphosphorylation of Tau not only causes defects in mitochondrial transport but also disrupts mitochondrial distribution and localization in mouse and cellular AD models (Cheng and Bai, 2018). It is reported that in tauopathies and AD, the integrity of microtubules is damaged via hyperphosphorylation and binding with protein Tau (Misko et al., 2010). Moreover, mitochondrial transport motor proteins and Tau (Ser199, Ser202, Thr205) are phosphorylated, which can lead to the dysfunction of mitochondrial transport in AD, mediated by a serine/threonine protein kinase, Glycogen synthase kinase 3 (Shahpasand et al., 2012). The aberrant aggregation of Tau is also demonstrated to induce microtubule instability, aggravating the impaired transport.

Amyloid-β (Aβ) oligomers and neurofibrillary tangles also elicit mitochondria dysfunction and oxidative stress (Butterfield and Boyd-Kimball, 2018; Mata, 2018), which may, in turn, impair axonal transport. It is demonstrated that oxidative stress is one of the typical event correlated with axon degeneration at the early stage of AD (Alavi Naini and Soussi-Yanicostas, 2015). Oxidative stress triggered by breaking the equilibrium between antioxidants and pro-oxidants may induce the excessive hyperphosphorylation of Tau. The aggregation of Tau is reported to inhibit microtubule transport, resulting in the decrease of peroxisome, which increase the incidence of oxidative stress (Stamer et al., 2002). Oxidative stress may in turn impair axon transport, which will aggravate tau phosphorylation in animal models and neuronal cultures of AD (Melov et al., 2007; Su et al., 2010). Interestingly, antioxidative treatment conducted in 3xTg-AD mice is found to alleviate oxidative stress, Tau hyperphosphorylation (Clausen et al., 2012). Therefore, the interconnection between oxidative stress and tau hyperphosphorylation seems to act a key role in axon degeneration of AD. Accumulating damaged mechanisms summarized above lead to the damage of axon homeostasis and finally, axonal degeneration in AD, which seems to provide some novel mitochondria-targeted therapeutic strategies in future.

### Parkinson's Disease

Parkinson's disease is a common late-onset neurodegenerative disease characterized by the degenerative death of dopamine (DA) neurons in the substantia nigra region and the formation of Lewy bodies, cytoplasmic inclusion bodies that contain α-synuclein (α-Syn) (Gao et al., 2018). Mitochondrial dysfunction is an early event in PD. Indeed, some familial PD cases are caused by mutations in genes encoding mitochondria proteins (e.g., Pink, Parkin, and DJ-1) (Cheng et al., 2010).

The degeneration of DA neurons in PD originates from the distal axons of fragile neurons which is long and highly branched (Surmeier et al., 2017). The increased size of axonal arborization which resulted in elevated mitochondrial bioenergetics is considered of the reason for the vulnerability for DA neurons in the substantia nigra (Pacelli et al., 2015; Giguere et al., 2019). Highly branched axons needs increased energy to deal with protein delivery, oxidative stress and mitochondrial dysfunction (Bhaskar et al., 2020).

Dysfunction of mitochondrial biogenesis plays a key role in the pathogenesis of PD. For instance, the inactivation of Parkin, which plays an important role in the pathological changes of familial PD, suppresses PCG-1α, a key regulator of mitochondrial biogenesis, ultimately causing the loss of DA neurons (Lee et al., 2017). In contrast, neurodegeneration caused by α-Syn overexpression in zebrafish can be partially relieved by PCG-1α upregulation (O'Donnell et al., 2014). Given that the dopaminergic axons loss precedes the cell death in both PD patient and PD model constructed by exposure to rotenone, a complex I inhibitor linked to PD (Tagliaferro and Burke, 2016), mitochondrial homeostasis in distal axon may participate in initial changes resulting in later neuron death. The limited early increase in mitochondrial density induced by rotenone in distal axons is due to upregulated distal axonal mitochondrial biogenesis (Van Laar et al., 2018), which is likely a compensatory process to mitochondrial disruption in PD.

Analysis of the post-mortem brains of PD patients showed that the distance between the synaptic terminal and mitochondria in DA neurons increased with a decreased volume and number of mitochondria, suggesting that mitochondrial synaptic availability and biomass contribute to degenerative neurons (Mallach et al., 2019). Mutations in Parkin impair Complex I activity, mitochondrial fusion, and the plasticity of synapses (Goldberg et al., 2003). Another example of how dysfunction mitochondria act in PD by disrupting synaptic plasticity comes from DJ-1, which is located in mitochondria and plays a role in synaptic transmission (Wang et al., 2008; Yan et al., 2020b).

Aberrant axonal transport contributes to neurodegeneration in PD (Lamberts et al., 2015). PD can be induced in mice by treatment with 6-hydroxydopamine (6-OHDA), which leads to axonal degeneration in dopaminergic neurons by inducing dysfunction of mitochondrial transport and microtubule disruption (Fuku, 2016). Significant alterations in mitochondria motility were observed in neurons induced from pluripotent stem cells (iPSCs) derived from an *LRRK2* mutant PD patient (Cooper et al., 2012). Prots et al. (2018) discovered that this alteration was associated with α-Syn oligomerization resulting from an increased α-Syn dosage, which resulted in reduced Miro and kinesin light chain-1 levels in axons and increased levels of Tau in neuronal soma.

PINK1 and Parkin are two proteins that can selectively remove damaged mitochondria. Mutations in these proteins can lead to PD and link mitophagy with this disease. Knockout of *Atg5* or *Atg7* causes loss of autophagy, resulting in swelling at the axon terminals and eventually cell death (Maday, 2016). Thus, autophagy plays an indispensable role in axon homeostasis. The accumulation of α-Syn and LRKK2 results from the loss of autophagy (Friedman et al., 2012), providing further proof that defects in autophagy cause the accumulation of abnormal organelles in axons (e.g., mitochondria), leading to oxidative damage and apoptotic cascades. In addition to PINK1/Parkin, LRRK2 is involved in initiating mitophagy by forming a complex with Miro. In pathologic *LRRK2G2019S* neurons, this function is disrupted but can be rescued by reducing Miro levels (Hsieh et al., 2016). While mitophagy has a protective effect on neurons, determining whether increased autophagy levels are the cause or effect of PD requires further study.

### Amyotrophic Lateral Sclerosis

Amyotrophic lateral sclerosis (ALS) is the most common adult motor neuron disease with characteristic progressive motor neuron degeneration, leading to muscle atrophy, paralysis, and ultimately death (Taylor et al., 2016). Approximately 90% of cases are sporadic (sALS), while the remaining 10% are familial (fALS) (Turner et al., 2017). fALS is largely associated with gene mutations, particularly in TAR DNA Binding Protein 43 (TDP-43) and Cu/Zn superoxide dismutase (SOD1) (Chia et al., 2018). Accumulating evidence suggests that axon degeneration in fALS is significantly correlated with mitochondrial damage, impairment of mitochondria transport, and autophagy-lysosomal dysfunction. Within an early stage in *SOD1G93A* mice, distal axonal transport is reported to be impaired, meanwhile, the expression of kinesin and dynein is also inhibited (Warita et al., 1999). In both *SOD1* patients (Boillée et al., 2006; De Vos et al., 2007) and *SOD1G93A* mice (Fischer et al., 2004; Damiano et al., 2006; Tallon et al., 2016), damaged mitochondria accumulate at distal sites, causing ATP deficiencies and aberrant calcium homeostasis at neuromuscular junctions, ultimately resulting in distal axon degeneration.

Enhancing mitochondria motility can remove the dysfunctional mitochondria from distal synapses meanwhile deliver the healthy ones to distal axons. Nevertheless, in motor neurons from *hSOD1G93A* mice, axonal mitochondria transport is damaged, and the defective mitochondria aberrantly accumulate in distal axons (De Vos et al., 2007; Magrané and Manfredi, 2009; Bilsland et al., 2010; Cozzolino et al., 2013). In order to address this defection, a research designed to cross *hSOD1G93A* and *SNPH*^−/−^ mice, aiming to examine whether enhancing mitochondria transport could make effect on the *hSOD1G93A* mice (Zhu and Sheng, 2011). Although the total mitochondrial transport is increased in the crossed mice, it seems to have no differences in the disease course. Moreover, this study reveals that deficits in mitochondrial transport appears to be insufficient to cause axon degeneration in the fALS-linked motor neurons (Zhu and Sheng, 2011), supported by an *in vitro* study in mutant *hSOD1* models (Marinkovic et al., 2012). In the motor neuron of fALS-linked *SOD1G93A* mice and the axons of AD-related cortical neurons from mutant human APP-expressing transgenic mice, impaired mitochondria are removed via the bulk release of SNPH cargo vesicles, which are mitochondria-derived and Parkin-independent, promoting mitochondria transport (Lin et al., 2017a). Recently, a study demonstrated that as the disease progresses in fALS-linked *SOD1G93A* and AD-linked mice, the quantity of SNPH cargo vesicles is also altered (Cheng and Sheng, 2020). It is interesting that at asymptomatic stages of fALS and AD, the number of SNPH cargo vesicles is greatly elevated in the axons of motor neurons (Lin et al., 2017a). Nevertheless, at the onset and late stages of the disease, SNPH is significantly depleted via mitochondrial impairment in these axons (Lin et al., 2017a). Furthermore, the study revealed that in response to these chronic pathological stresses, the quantity of SNPH cargo vesicles changes in distal axons. The defective mitochondria anchored by SNPH are transported to the soma for recovery or degeneration (Cheng and Sheng, 2020). The SNPH-mediated pathway acts as a protective mechanism during the early asymptomatic stages of fALS and AD and is independent of and acts before PINK1/Parkin mediated mitophagy (Lin et al., 2017b). Thus, it is regarded as a significant hallmark of diagnosis and an early target for treatment. However, simply increasing mitochondrial transport by turning off SNPH-mediated anchoring (Lin et al., 2017a) or eliminating SNPH (Zhu and Sheng, 2011) fails to replenish ATP deficiency in *hSOD1G93A* mutant mice. The autophagy-lysosomal function is also defective in fALS-linked mice at an early stage of disease both *ex vivo* and *in vivo*, preventing the elimination of damaged mitochondria from distal axons (Xie et al., 2015). Thus, the combination of enhanced mitochondria transport and increased defective mitochondria elimination may be an effective therapeutic strategy for fALS.

A study has reported that anterograde and retrograde transport of mitochondria in primary motor neurons axons are greatly defected via overexpression of wild-type *TDP-43*. Interestingly, the study demonstrated that the loss of *TDP-43* also inhibits axonal transport of mitochondria, revealing that damaged mitochondria axonal transport may involve various pathways mediated by *TDP-43* (Wang W. et al., 2013). Moreover, a recent study showed that specific protein synthesis is inhibited (i.e., transcripts involved in mitochondrial energy metabolism, cytoskeletal components, and translational machinery) within the axons of motor neurons in *TDP-43*-knockout mice (Briese et al., 2020). Thus, axonal transport and mitochondria function are significantly defected in *TDP-43*-knockout mice, leading to the impairment of axon growth as well as revealing that depletion of *TDP-43* may act a predominant role in axon degeneration of ALS (Briese et al., 2020).

### Treatment

In neurodegenerative diseases, the degeneration of axons often occurs at early disease stages. This degeneration is often accompanied by the accumulation of damaged or fragmented mitochondria. Therefore, maintaining mitochondrial dynamics in axons is a common treatment strategy.

Amyloid-β (Aβ) toxicity and Tau dysfunction in AD, often accompanied by impaired axonal mitochondrial transport, jointly lead to disease progression. Wang et al. (2019b) demonstrated that mitochondrial transport defects can be corrected in Aβ-treated neurons by protecting KIF5A, suggesting that maintaining normal mitochondrial axon transport is a valuable treatment strategy in AD. There are many targeted treatments for reducing the mitochondrial content caused by defective mitochondrial trafficking, as represented by increased retrograde transport rates in axons (Schwab et al., 2017). In addition, glycolytic defects in oligodendrocytes induce axon degeneration in both AD mice and patients via the Drp1-hexokinase 1-NLRP3 (NLR family pyrin domain containing 3) signaling axis, which is considered to be a therapeutic target (Yan et al., 2020a; Zhang et al., 2020). Mdivi-1, Dynasore, and P110 are promising agents that maintain axonal mitochondrial turnover by inhibiting dynamin to prevent the production of fragmented mitochondria, maintain normal mitochondria morphology, and restore ATP levels in a PD model (Elfawy and Das, 2019). Additionally, deep brain stimulation (DBS) is a very effective intervention to treat patients with advanced PD and its mechanism is related to the restoration of the number and volume of mitochondria (Mallach et al., 2019). The selective peptide inhibitor, P110 inhibits the Drp1-Fis1 interaction and improves the structure and function of mitochondria in *G93A SOD1* mutant mice, indicating an attractive target for ALS patients (Joshi et al., 2018). Nicotinamide mononucleotide adenylyl transferase1 (Nmnat1), an enzyme involved in nicotinamide adenine dinucleotide (NAD^+^) synthesis, has a protective effect on mitochondrial dynamics and morphology following vincristine-induced axon degeneration (Berbusse et al., 2016).

Reactive oxidative species (ROS) production caused by mitochondrial dysfunction leads to oxidative stress, an important factor in axonal degeneration. Therefore, many treatment strategies focus on mitigating local ROS production using novel antioxidants that directly target mitochondria. Some mitochondria-targeted compounds (e.g., latrepirdine, methylene blue, triterpenoids, and the mitochondrial-targeted coenzyme Q10 derivative MitoQ) have been extensively evaluated in *in vivo* and *in vitro* AD and PD models (Elfawy and Das, 2019). In addition, a significant reduction in ROS levels results from treatment with the selective peptide inhibitor P110 and improves motor capacity and survival rates in G93A SOD1 mutant mice (Joshi et al., 2018).

Axon regeneration is an attractive target in neurodegenerative diseases. Because growth cone formation requires a large ATP supply, targeted mitochondrial therapy is key to axonal regeneration. While mature CNS neurons almost lose their intrinsic capacity for axon regeneration, upregulation of axon cytoskeleton proteins was observed in the 6-OHDA-hemiparkinsonian rat model, indicating a high plasticity and regeneration potential in adult animals (Lessner et al., 2010).

The *G2019S* mutation in the conserved serine kinase MAPK kinase domain contained in LRRK2 is the cause of familial PD (Cookson, 2010). Therefore, reduced neurite outgrowth and increased growth cone size were observed in neurons of *LRRK2 G2019S* mutant mice and the level of F-actin in growth cone also increases (Parisiadou et al., 2009), suggesting that growth cone formation, an essential step for axon regeneration is seriously impacted in PD. However, in the 6-OHDA-induced PD animal model, there are many compensatory responses in early-stage PD, including the upregulation of some proteins related to axon regeneration. Dihydropyrimidinase-related protein 2 (DPYSL2), which is important for axon outgrowth and regeneration (Sun and Cavalli, 2010), first decreased and then gradually recovered in this model. Moreover, axon growth and mitochondrial transport-related proteins (e.g., dynein, dynamin, and myosin) were also overexpressed (Kuter et al., 2016). Thus, a potential therapy to promote axon regeneration would be to enhance the expression of mitochondrial transport-related proteins. A new promising therapeutic method involving exogenous mitochondrial transplant effectively attenuates the progression of neurologic disorders, including experimental Parkinson's disease (Shi et al., 2017). Supportive mitochondrial-targeting therapy is a promising approach for future therapeutic intervention in the CNS to promote axon regeneration. However, it should be noted that there are currently very limited ways to treat neurodegenerative diseases by promoting axon regeneration because of the diminishing intrinsic axonal regeneration in mature CNS neurons compared to PNS neurons, and there are no optimal treatments for these neurodegenerative diseases. Thus, researchers have focused on stopping the degeneration of these nerves. With the deepening understanding of the treatment of mitochondria to prevent neural degeneration, we believe that axonal mitochondria regeneration will become a better therapeutic target for treating neurodegenerative diseases.

## Discussion

**Figure 1:**
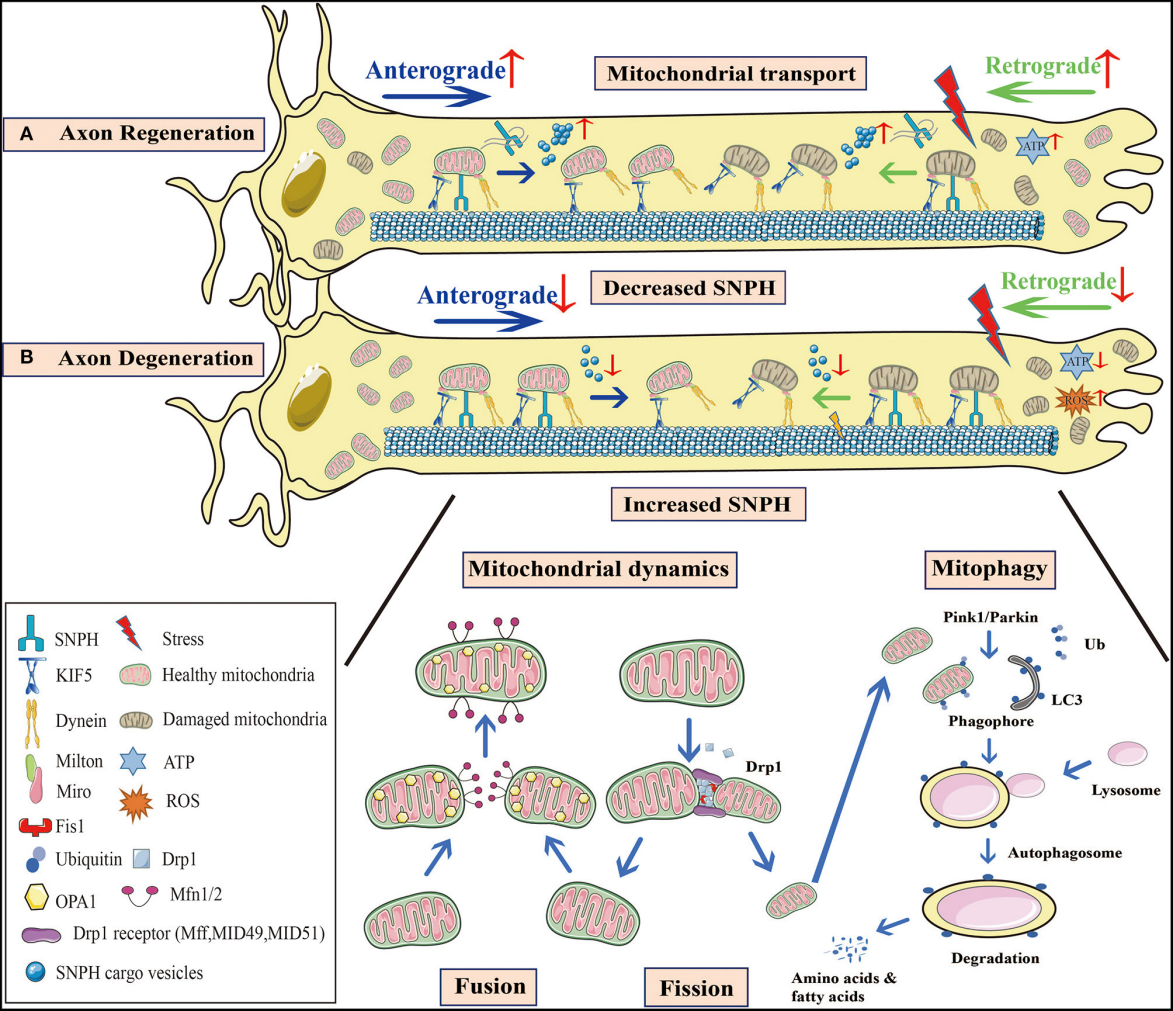
Mitochondrial behavior in axon degeneration and regeneration. **(A)** Axon regeneration: Since this process requires considerable energy, mitochondrial density at distal axons is elevated to provide sufficient ATP. Not only is anterograde transport of healthy mitochondria promoted, but so too is retrograde transport of damaged mitochondria, to increase mitochondrial density at regenerative zones. SNPH knockout in TBI models, or the bulk release of SNPH cargo vesicles at AD and fALS early stages, both enhance axonal mitochondrial transport. **(B)** Axon degeneration: Mitochondrial dysfunction and oxidative stress occur in injured axons. When axonal mitochondrial transport is seriously impaired, damaged mitochondria aggregate at distal zones and the healthy ones fail to transport from the proximal zones to replenish ATP insufficiency. A decrease in SNPH cargo vesicle release at later stages of AD and fALS, as well as increased SNPH expression in SCI models inhibit mitochondrial transport. Moreover, axonal microtubule loss contributes to impaired axonal transport in TBI. Mitochondrial dynamics are also defective during axon degeneration. Due to increased Drp1-mediated fission in SCI, mitochondrial fragmentation is aggravated, causing mitochondrial damage. These organelle fuse with healthy mitochondria, however, most undergo mitophagy. In PD, Pink1-Parkin dependent mitophagy is defective, facilitating damaged mitochondrial accumulation and apoptotic cascades. These processes are interconnected in response to stress, and collectively lead to axon degeneration. SNPH, syntaphilin; TBI, traumatic brain injury; AD, Alzheimer's disease; fALS, familial amyotrophic lateral sclerosis; SCI, spinal cord injury; PD, Parkinson's disease.

Axon degeneration and regeneration are essential parts of CNS neuronal death and restoration of CNS neurons, respectively, in aging, injury, and neurodegenerative diseases. However, the decline in the intrinsic regrowth capacity of mature CNS axons leads to a failure to regenerate after neuronal impairment (Fawcett and Verhaagen, 2018). Accumulating evidence demonstrates that mitochondrial dysfunction is intimately correlated with the initiation of axon degeneration and inhibition of axon regeneration (Qian and Zhou, 2020). Despite the mechanism underlying is still elusive, it suggests that mitochondrial quality control is an important intrinsic capacity of axon regeneration. For axon degeneration, mitochondrial dysfunction not only causes energy deficits and oxidative stress but also comprises mitochondrial dynamics, axonal transport, and mitophagy (Court and Coleman, 2012; Vasic et al., 2019). In contrast, restoring and enhancing these processes during axon regeneration increases the capacity of intrinsic regrowth (Figure 1). It comes to a consensus that axon regeneration requires adequate energy production and mitochondrial transport both in aging and in diseases (Zhou et al., 2016; Sheng, 2017; Zheng et al., 2019). Defected mitochondria can be replaced via mitochondria injection, which is demonstrated to decrease oxidative stress and trigger axon regeneration both *in vitro* and *in vivo* (Kuo et al., 2017). Thus, further study of axonal mitochondrial behavior under these conditions is important, not only for the alleviation and treatment of axon degeneration but also for identifying potential targets to enhance axon regeneration.

It is generally accepted that diminished intrinsic regeneration is a major barrier for axon regeneration in mature CNS neurons. Analysis of intrinsic axon degeneration and regeneration pathways and signaling networks can bring new insights for potential mitochondria-targeted therapeutic strategies. For example, a novel therapeutic strategy for enhancing the intrinsic capacity of axon regeneration may include genetic reprogramming through epigenome modifications and the transcriptome (Qian and Zhou, 2020). Mitochondrial damage-associated molecular patterns (mtDAMPs) released by injured axonal mitochondria can activate Schwann cell processes mediated by formylpeptide receptor 2 (FPR2) and toll-like receptor 9 (TLR9), which have a pivotal role in axon regeneration and cell migration (Korimová et al., 2018). Therefore, regulation of these mitochondria-targeted genetic reprogramming can be considered to be a promising molecular strategy for activating axon regeneration. At the cellular level, enhancing mitochondrial transport could greatly contribute to axon regeneration by removing defective mitochondria and replenishing healthy mitochondria at injured axon sites (Zheng et al., 2019). In addition, glial cells (astrocytes, oligodendrocytes, and microglia) contribute to mitochondrial dysfunction during axon growth inhibition and regeneration failure in the adult CNS (Brosius Lutz and Barres, 2014; Cregg et al., 2014; Silver and Silver, 2014). Astrocytes inhibit axonal mitochondrial energy metabolism by increasing nitric oxide production, glutamate levels, and intracellular calcium influx during Multiple Sclerosis (MS) (Correale and Farez, 2015). Similarly, astrocytes can release mitochondria containing particles, and transport them to defective axons following stroke (Babenko et al., 2015; Hayakawa et al., 2016). *In vitro* astrocyte-to-neuron mitochondrial delivery and *in vivo* astrocyte-derived mitochondrial transport improves neuronal survival, plasticity, and behavior outcomes (Hayakawa et al., 2016). Reactive oxygen and nitrogen species generated by microglia directly damage neurons by inhibiting cytochrome C oxidase and mitochondrial respiratory chain complex IV, causing axonal mitochondrial dysfunction in MS (Nikić et al., 2011). Furthermore, oligodendrocytes and astrocytes provide axonal mitochondria with pyruvate or lactate, which are imported into the mitochondria for energy metabolism during MS and AD (Fünfschilling et al., 2012; Correale et al., 2019; Zhang et al., 2020). Clearance of damaged mitochondria not only relies on mitophagy at the injury sites but also depends on mitochondrial retrograde transport, which involves SNPH and dual leucine zipper kinase 1 (Han et al., 2016; Cheng and Sheng, 2020). However, many molecules and substrates engaged in mitochondrial transport or dynamics need to be clarified. Moreover, to strengthen the intrinsic capacity for regrowth, improving the extrinsic inhibiting environment could make a critical difference in axon regeneration. Drugs targeted at oxidative stress via the clearance of ROS (Geden and Deshmukh, 2016) or directed at the restoration of calcium homeostasis by inhibiting calcium channels and calcium-activated enzymes (Mu et al., 2015) will aid axon regeneration. Furthermore, remodeling the neuronal cytoskeleton may be a novel mechanism to alleviate the extrinsic inhibitory cues (Qian and Zhou, 2020). In conclusion, combined approaches that target mitochondria, which increase the intrinsic capacity and decrease the extrinsic inhibiting environment, may provide an effective therapeutic strategy to enhance axon regeneration in aging, injury, and neurodegenerative diseases.

## Author Contributions

BW, MH, BZ, and XZ made substantial contributions to the conception and design of the study. BW, MH, DS, XY, BZ, and XZ participated in drafting the article. BW and MH created the figure. All authors contributed to the article and approved the submitted version.

## Conflict of Interest

The authors declare that the research was conducted in the absence of any commercial or financial relationships that could be construed as a potential conflict of interest.
